# miR-34a: A Promising Target for Inflammaging and Age-Related Diseases

**DOI:** 10.3390/ijms21218293

**Published:** 2020-11-05

**Authors:** Angela Raucci, Maria Cristina Vinci

**Affiliations:** 1 Unit of Experimental Cardio-Oncology and Cardiovascular Aging, Centro Cardiologico Monzino-IRCCS, Via Carlo Parea 4, 20138 Milan, Italy; 2 Unit of Vascular Biology and Regenerative Medicine, Centro Cardiologico Monzino-IRCCS, Via Carlo Parea 4, 20138 Milan, Italy

The term “inflammaging” describes the chronic, low-grade systemic inflammation that occurs during physiological aging in the absence of an overt infection [1,2]. Inflammaging is a hallmark of all age-related diseases, including cardiovascular diseases (CVDs) and associated risk factors (e.g., diabetes), that affects morbidity and mortality in the elderly [1,2]. Along with specialized immune cells, senescent cells are recognized as the largest contributors to inflammaging thanks to the acquisition of a senescence-associated secretory phenotype (SASP) that enable them to secrete a variety of soluble molecules including proinflammatory cytokines and chemokines, growth factors and matrix degrading proteins [2,3]. Furthermore, senescence may promote cell trans-differentiation toward a pathological phenotype [4]. Vascular calcification (VC) is an age-related complication of atherosclerosis, type 2 diabetes mellitus (T2DM) and chronic kidney disease characterized by the transition of vascular smooth muscle cells (VSMCs) to an osteo-chondrogenic phenotype with consequent hydroxyapatite crystals deposition and mineralization of the arterial wall [5,6]. Senescent VSMCs have greater propensity to experience the osteoblastic switch since express bone-related genes, such as Runt-related transcription factor 2 (Runx2), alkaline phosphatase and osteocalcin and secrete pro-calcification SASP factors, like interleukin 6 (IL-6), bone morphogenetic protein 2 and osteoprotegerin responsible for the spreading of senescence and mineralization of neighboring VSMCs [7,8].

MicroRNA-34a (miRNA-34a) is a senescent-associated miRNA whose expression has been shown to increase in different tissues and organs with age [9,10]. miR-34a is a promoter of senescence-induced VC. *Mir34a* deletion in mice reduces the expression of the VC markers such as SRY (sex-determining region Y)-box 9 (Sox9) and Runx2 and senescence proteins p16 and p21 and, consequently soft tissue and aorta medial calcification [4]. In vitro, replicative senescent human aortic smooth muscle cells (HASMCs) show higher levels of miR-34a and its ectopic overexpression in proliferative cells induces growth arrest and senescence through direct downregulation of AXL receptor tyrosine kinase (Axl) and sirtuin 1 favoring HASMCs mineralization in hyperphosphatemia conditions [4,9].

Our recent work, published in the Special Issue “Mechanisms of Inflammation in Degenerative Cardiovascular Conditions 2.0” of this journal, demonstrates that miR-34a enhances the secretion of several SASP factors in HASMCs, such as pro-inflammatory molecules (IL6, IL12, IL13 and Growth-Regulated Oncogene-alfa (GRO-α)), the metalloprotease inhibitor TIMP2 and the Insulin-like Growth Factor Binding Protein 3 (IGFBP3) [11]. Preconditioning with miR-34a-induced “secretome” enhances HASMCs senescence and mineralization indicating that this miRNA is able to endorse the activation of the VSMCs SASP to fuel the inflammatory conditions responsible for the spreading of vascular cells senescence and calcification [11]. Accordingly, *Mir34a* genetic ablation prevents the induction of IL6 expression occurring during aortas medial calcification onset. Importantly, we also found a positive correlation between circulating miR-34a and IL6 in a population of healthy subjects spanning from 20–90 years [11]. Altogether, our findings pinpoint miR-34a as a driver of vascular and systemic low-grade inflammaging and, hence, a causal promoter of age-associated diseases.

T2DM shares a number of important features with aging, including inflammaging and VC [12,13]. Indeed, high glucose triggers numerous inflammation and endoplasmic reticulum (ER) pathways that contribute to VSMCs senescence and calcification [13,14]. To date, there is strong evidence that diabetic milieu may epigenetically skew CD34^+^ stem cell differentiation, a cell population endowed of both regenerative and hematopoietic properties, towards more inflammatory cell populations [15]. To this regard, clinical and preclinical studies described abnormal elevation of monocyte subsets with higher inflammatory phenotype, alteration in macrophage polarization, as well as in the levels of circulating cytokines [16,17,18]. Interestingly, a very recent study demonstrated that non-classical monocytes exhibit the hallmarks of senescence, suggesting that their pro-inflammatory nature could be the manifestation of SASP [19]. In the Special Issue “Bone Marrow and Stem Cell Alterations in Diabetes: Causes, Consequences and Therapeutics” of this journal we recently reviewed the pathological contribution of bone marrow (BM) stem cells to diabetic cardiovascular complications [20]. In particular, we described the ability of diabetic milieu to redirect stem cell differentiation into cell populations with calcifying phenotype (osteoprogenitor cells) [21]. These cells, hypothetical “side products” of differentiation drift, witness the ability of diabetes to promote the generation of cells with pro-calcifying properties among others [20], with clear implications in diabetic micro- and macro-angiopathies development. Up to now, few preclinical studies reported that miR-34a up-regulation in the diabetic context impairs vascular function [22]; however, there are no data regarding the involvement of this miRNA in the processes of VC and inflammation associated with T2DM.

Since miRNAs are emerging as promising druggable targets, extending the knowledge of the mechanisms by which miR-34a regulates cell senescence, trans-differentiation and SASP acquisition in different pathological contexts, will help to develop new pharmacological therapies to counteract inflammaging and, eventually, age-related diseases onset.
